# DeStripe: frequency-based algorithm for removing stripe noises from AFM images

**DOI:** 10.1186/1472-6807-11-7

**Published:** 2011-02-01

**Authors:** Shu-wen W Chen, Jean-Luc Pellequer

**Affiliations:** 1 CEA, iBEB, Service de Biochimie et Toxicologie Nucléaire, F-30207 Bagnols sur Cèze, France

## Abstract

**Background:**

Atomic force microscopy (AFM) is a relatively recently developed technique that shows a promising impact in the field of structural biology and biophysics. It has been used to image the molecular surface of membrane proteins at a lateral resolution of one nanometer or less. An immediate obstacle of characterizing surface features in AFM images is stripe noise. To better interpret structures at a sub-domain level, pre-processing of AFM images for removing stripe noises is necessary. Noise removal can be performed in either spatial or frequency domain. However, denoising processing in the frequency domain is a better solution for preserving edge sharpness.

**Results:**

We have developed a denoising protocol, called *DeStripe*, for AFM bio-molecular images that are contaminated with heavy and fine stripes. This program adopts a divide-and-conquer approach by dividing the Fourier spectrum of the image into central and off-center regions for noisy pixels detection and intensity restoration; it is also applicable to other images interfered with high-density stripes such as those acquired by the scanning electron microscope. The denoising effect brought by *DeStripe *provides better visualization for image objects without introducing additional artifacts into the restored image.

**Conclusions:**

The DeStripe denoising effect on AFM images is illustrated in the present work. It allows extracting extended information from the topographic measurements and implicitly enhances the molecular features in the image. All the presented images were processed by *DeStripe *with the raw image as the only input without any requirement for other prior information. A web service, http://biodev.cea.fr/destripe, is available for running *DeStripe*.

## Background

Unlike other optical-based microscopes, atomic force microscope (AFM) is a sensing instrument [1]. In brief, a nano-sized tip located beneath a micro-cantilever scans across a field of deposited molecules. The cantilever deflection can be detected by a laser beam that reflects off the back of the cantilever. With a set of piezoelectric ceramics connected to the cantilever, the so-called height image of molecules can be made at a constant applied force. Because of its exceptional high signal/noise ratio, AFM is able to measure the topography of a single isolated molecule with a lateral resolution of a few nanometers and a vertical resolution of a few Angstroms [2].

Acquisition of high-resolution images of macromolecules in aqueous solution using AFM does not require sample staining [3]. Development of AFM imaging techniques in life sciences is progressing [4] including imaging single isolated molecules at high speed [5]. To date very high-resolution imaging by AFM has been obtained on membrane proteins 2 D crystals [6,7] as well as on densely packed proteins in native membranes [8-10]. Although AFM is primarily an imaging tool, it also allows measuring inter- and intra-molecular interactions on the pico-Newton scale [11-14] and force-probing the surfaces of living cells at the single molecule level [15,16] in order to map protein receptors for example[17].

Image quality is highly involved in feature interpretation and extraction for sampled objects in all kinds of imaging systems. Noise is a critical artifact that influences image quality and is mainly produced during image acquisition. It needs to be removed or reduced for further data processing to acquire desirable information or feature interpretation. There is no exception for AFM images. Among all types of noises, stripe noise is the notorious one that profoundly degrades image quality acquired by AFM, and is a consequence of the scanning pattern. During the scanning along one line of image, the sample surface height is acquired by an oscillating motion of the cantilever tip in the perpendicular direction to the substrate plate. Stripe noise may occur, for example using the tapping mode of AFM [18], from a loss or inadequate acquisition of height information. Any abrupt increase of the force from samples exerted on the tip would make a dramatic change in the tip vibration such that the noise cannot stay constant during the scanning; moreover these noise errors cannot be averaged off. A critical factor causing this change is the interaction between the sample and the tip. Thereby, these stripes vary in intensity, length, local density and frequency range, subjective to a variety of factors, such as sample preparation conditions and the constituents therein on the substrate plate. In particular, sharp and irregular boundaries or protuberances in the object distribution usually produce serious stripes across the image.

Noise is usually modeled as an additive term in the intensity distribution with a Gaussian or Poisson form of zero mean and constant variance [19]. This assumption becomes inapplicable if the image intensity is non-randomly distributed. From the noise origin described above, the high-density and fine stripes observed in AFM images do not occur randomly (neither in time nor in space). An image frequency spectrum can depict the noise characteristics [20]. In traditional strategies of Fourier transform, a filter with high or low pass is often set up to gate the frequencies composed of stripe noises. However, this method sometimes removes some image details when stripe noises are mixed with components from object textures. Recently, a combination of wavelet and Fourier transform has been developed to remove both stripe and ring artifacts from images [21]. Alternatively, a method based on the heterogeneity of image frequency spectrum has been proposed to remove periodic and quasi-periodic stripes [22]. However, as shown in this paper, the stripe noises observed in AFM images are not in a unique or specific form. Although we adopted a similar approach for developing the denoising protocol, some new designs are highlighted for the removal of non-uniform and high-density stripe noises from AFM images.

## Implementation

To be visualized by the human eye, the denoising procedure was not performed straightforwardly on the spectrum amplitude but on its logarithmic scale, the corresponding image of which is called LogF. We proposed a divide-and-conquer strategy to proceed the denoising in two separate regions of the frequency domain, i.e., the central and the off-center regions, as most of the high intensities are concentrated in the neighborhood of the origin. In order to best preserve the original data and reduce the computational task, we employed a decision-based algorithm [23] to select pixels for the variance test [24] and intensity reconstruction. Heterogeneity measurements were performed for detecting a spectral pixel potentially responsible for stripe noise in the raw AFM image.

**Figure 1:**
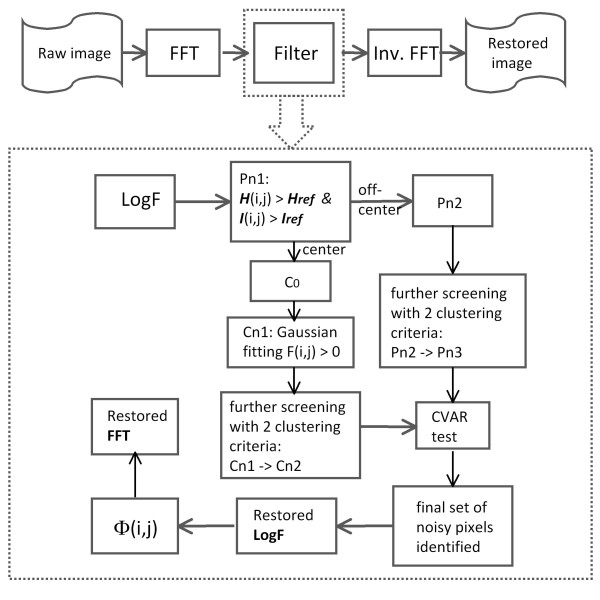
**The flow chart of *DeStripe*, see details in Implementation**. The dash-lined box describes the steps of reducing the number of potentially noisy pixels. The denotation of pixel sets at various steps is stated in Implementation.

Regarding the image restoration, the intensity replacement was also performed in the image spectrum, and the processed image was obtained by the inverse Fourier transformation. In general, a smoothing procedure applied in the image spatial domain can be exactly used in its frequency domain, e.g., a median-like filter [22]. Yet, processing in the frequency domain is a better approach than in the spatial domain for that the edge sharpness can be better preserved. Moreover, we attempted choosing a size for the local window to restore the image intensity as small as possible. Consequently, the accuracy of image restoration is only jeopardized at a minimal degree in the case where pixels are falsely picked up as noisy. The design of our denoising protocol is outlined in Figure 1 and details are described below.

### Heterogeneity function

The heterogeneity of LogF determines whether a pixel is noisy or not. The heterogeneity measurement ranges from 0 to 1; the larger the value, the more heterogeneous is the intensity. There are two components in the heterogeneous function, i.e., abrupt change in intensity and intensity itself. The former is represented by the Laplacian of LogF. Table 1 presents the discrete Laplacian operator used in *DeStripe*. For each component, the values were offseted and normalized over the entire image such that they are confined within [0, 1]. Accordingly, the heterogeneity function *H *at the pixel (*i, j*) is expressed as

**Table 1:** The discrete Laplacian operator used in *DeStripe*.

-1	-1	-1
-1	8	-1

-1	-1	-1

$$H(i,j)=\frac{L(i,j)-L_{\min}}{L_{\max}-L_{\min}}\times \;\frac{I(i,j)-I_{\min}}{I_{\max}-I_{\min}},$$

where *L*(*i, j*) and *I*(*i, j*) represent the Laplacian and intensity values at pixel (*i, j*), respectively, *L_min_*, *L_max_*, *I_min _*and *I_max _*correspond to their maxima and minima. As a result, the pixels with higher *H *values possess higher intensity and experience more dramatic change in intensity.

### Global sampling of pixels

A preliminary sampling of noisy pixels was done by thresholding the *H *value based on an internally determined value (*H_ref_*) extracted from the heterogeneity histogram which is analog to the homogeneity level in removal of impulse noises [25]. For convenience, we denoted this set of sampled pixels as P_n_1 and the algorithm is described below:

1. Calculate *H *values of LogF.

2. Form the histogram of *H *with 20 bins such that the variation of *H *in each bin is within 0.05.

3. Find the most spreading group of consecutive bins with non-zero populations in the histogram.

4. From the most spreading group, find the threshold bin in the direction of increasing heterogeneity. The threshold bin is defined as the first bin encountered such that the ratio of its population relative to that of the most populated bin is ≤ 0.5.

5. Calculate the *H *threshold as *H_ref _*= 0.5 (*H_up _*+ *H_low_*), where *H_up _*and *H_low _*correspond to the upper and lower limits of the threshold bin.

6. Calculate the intensity threshold as *I_ref _*= 0.5 (*max_ref _*+ *ave_ref_*), where *ave_ref _*and *max_ref _*are the average and the maximum of intensities over the pixels whose *H *≤ *H_ref_*, respectively. A relevant parameter, variance (*var_ref_*), is also calculated.

7. P_n_1 = {(i, j)| *H*(i, j) >*H_ref _*&*I*(i, j) >*I_ref_*}.

### Divide-and-Conquer Strategy

Due to dramatic variations in intensity in the central region of the frequency domain, we divided P_n_1 into two groups; one was referred to as the central region and the other as the off-center region.

### Formation of the central region

The central region was considered as a circular disk. The initial radius of the disk was derived from the moment of inertia tensor of P_n_1, where the mass magnitude was replaced by the intensity value in the tensor array [26]. We calculated the eigenvalues (*σ_x_, σ_y_*) and eigenvectors (ê_x_, ê_y_) of the tensor array. The initial radius equals the square root of *σ_x _*+ *σ_y_*. Starting from the center of intensity distribution, (*i_0_, j_0_*), we expanded stepwise the region outwardly with an increment of 1/10 of the initial radius value. At each expansion step, we counted the P_n_1 pixels and the total within the newly expanded region; if the ratio of the two numbers was ≤ 0.85, then the expansion was stopped and all the visited pixels ∈ P_n_1 were included as members in the central region; we denoted it as C_0 _and P_n_1 - C_0 _as P_n_2.

### Sampling of noisy pixels in the central region

In order to avoid vain data treatments and reduce the number of false noisy pixels recruitment, we modeled the intensity distribution of C_0 _by an anisotropic Gaussian function. We used the Levenberg-Marquadt algorithm [27] to fit the intensity nonlinearly into the model function. In other words, we minimized the objective function,$\sum_{i,j}f{\left(i,j\right)}^{2}$, where *f *(*i, j*) was expressed as

$$f(i,j)=\frac{I(i,j)}{I(i_{0},j_{0})}-e^{\left\{-c_{1}\cdot \frac{{\left(i-i_{0}\right)}^{2}}{\sigma_{x}^{'}}-c_{2}\cdot \frac{{\left(j-j_{0}\right)}^{2}}{\sigma_{y}^{'}}\right\}},$$

where *I*(*i_0_, j_0_*), *c_1 _*and *c_2 _*are fitting parameters; *I*(*i_0_, j_0_*) is the restored intensity at (*i_0_, j_0_*), and *c_1 _*and *c_2 _*shape the anisotropic breadth of the Gaussian fitting; *I*(*i, j*) is the intensity at (*i, j*). The relationship between (*σ_x_', σ_y_'*) and (*σ_x_, σ_y_*) can be obtained through (ê_x_, ê_y_) as [28]

$\sigma_{x}^{'}=\sigma_{x}\times \cos^{2}\theta +\sigma_{y}\times \sin^{2}\theta$, and

$$\sigma_{y}^{'}=\frac{\sigma_{x}\times \sigma_{y}}{\sigma_{x}^{'}},$$

where *θ *is the angle that rotates counterclockwise the Cartesian x, y-coordinate axes aligned with (ê_x_, ê_y_). Note that (*σ_x_, σ_y_*) and (ê_x_, ê_y_) were computed based on the right-handed rule while (*σ_x_', σ_y_'*) were defined according to the convention of image presentation, i.e., the origin is located at the left-top corner of the image, the image *j *row and *i *column represent the x- and y-axes, respectively. For a pixel (*i, j*) ∈ C_0_, if *f *(*i, j*) ≤ 0, then the pixel was not considered as corrupted with noise, otherwise it was included in a set denoted by C_n_1.

We selected data points in C_n_1 with a 10-bin histogram using the same thresholding method as described previously for global sampling. The collected pixels were further screened and clustered based on two criteria: (1) if the pixel distribution in the form of horizontal or vertical line was > 2/3 of the region of interest; (2) if the length of consecutive pixels was ≥4. The qualified pixels with either condition were considered as noisy and formed the set, C_n_2. The same procedure was also applied to the off-center region, P_n_2; the resulting pixel set is denoted by P_n_3. Maps of these sets are presented in additional file 1 Figure S1, for each study image.

### Constrained variance (CVAR) test and intensity restoration

For a processed pixel (*i_c_, j_c_*), the local mean and variance for the CVAR test were calculated as:

ave=1Nvar∑i=−NSNS∑j=−NSNSI(i+i,cj+jc),

σvar=1Nvar∑i=−NSNS∑j=−NSNS(I(i+i,cj+jc)−ave)2.

The imposed constraint is that only non-noisy pixels were counted for *N_var _*within the (2*N_S_*+1) *× *(2*N_S_*+1) local window, i.e., (*i*+*i_c_, j+j_c_*) neither ∈ C_n_2 nor ∈ P_n_2. In the present work, *N_S _*= 1. Define$\sigma_{std}=\sqrt{\sigma_{\mathrm{var}}}$, if *I*(*i_c_, j_c_*) - *ave *>*σ_std_*, then *I*(*i_c_, j_c_*) was replaced by *ave*, otherwise the original *I*(*i_c_, j_c_*) was reserved. For P_n_2, the CVAR test was not performed point by point; instead, we clustered the connected pixels and the test was performed starting at the boundary pixels of each cluster.

### Filter function

The major component of *DeStripe *is the filter function that turns a noisy image into a clean one. Consider *S*(*i, j*) and *S*(*i, j*)' as the measured and restored intensity values at pixel (*i, j*) in the LogF image, respectively, then the filter function was calculated as $\Phi (i,j)=\frac{e^{S{\left(i,j\right)}^{'}}}{e^{S(i,j)}}$; the value range is (0, 1], i.e. 0 < Φ(*i, j*) ≤ 1. Accordingly, the restored and the associated noise images were obtained by the inverse FFT of the products of exp[*S*(*i, j*)] with Φ(*i, j*) and 1-Φ(*i, j*), respectively. The image formed by the Φ(*i, j*) values is henceforth called Φ-image.

## Results and Discussion

We present two biological systems of which the topographic images were measured by AFM. One contains biomembranes and the other is constituted of proteins belonging to a large family of hydrolase enzymes, GTPase. We applied *DeStripe *to these raw AFM images for stripe noise reduction. The main purpose of the denoising is to better reveal molecular features in images that are distinguishable to human vision. Lastly, the applicability of *DeStripe *was also tested for stripe removal from images acquired by a scanning electron microscope, SEM [29].

### Denoising vs. high-resolution AFM imaging

In high-resolution AFM imaging, one goal is to measure molecular topographies down to a sub-nanometer scale. In most occasions, one may seek an AFM tip as small as possible for probing the sample surface; the usual radius for AFM tips is 5-10 nm [30]. However, we found that the AFM image quality also profoundly depended on the study system. In other words, one set of instrumentation parameters may not guarantee to gain similar quality for images with different biological constituents, as the sample-related stripe noise is always a major hindrance.

**Figure 2:**
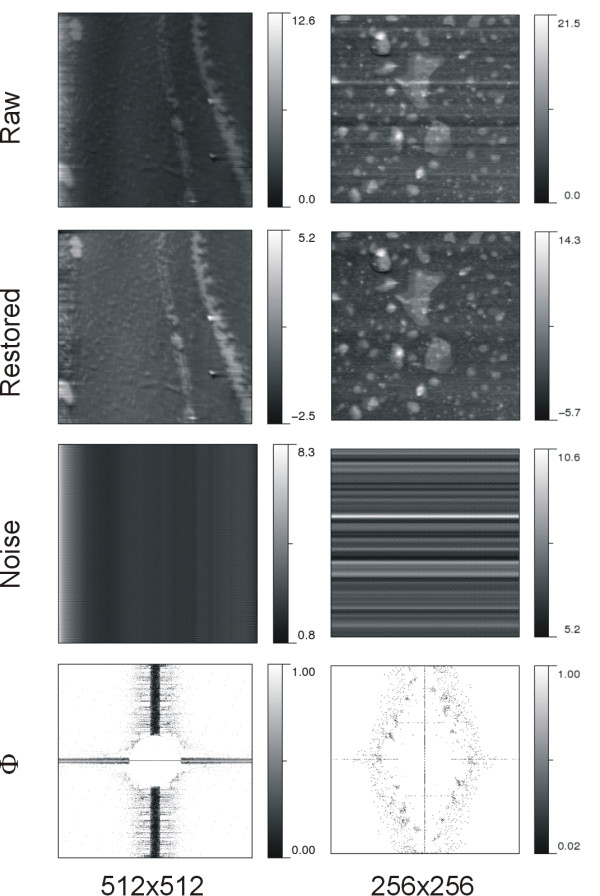
**AFM imaging on biomembranes**. Numbers at the bottom of the figure are the number of pixels composing the image. On the left column, the image area and the intensity unit are 10.0 × 10.0 μm^2 ^and 1.0 nm, respectively. On the right column, the image area is 2.0 × 2.0 μm^2 ^and the intensity is in the unit of 1.0 nm. Note that the varying shading degree in the calibration bar is used for inspecting intensity variations in the image that are bounded by the extreme values indicated.

**Figure 3:**
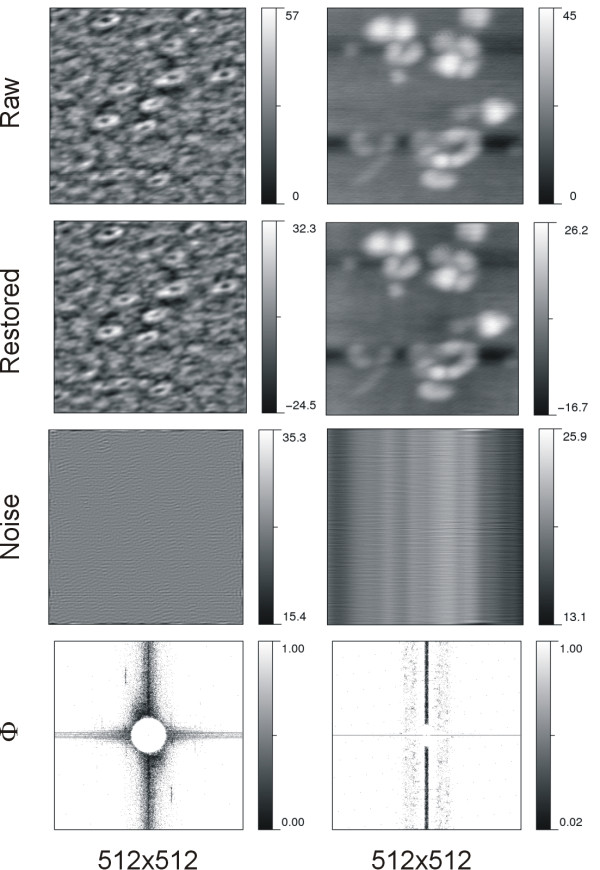
**AFM imaging on GTPase enzymes**. Numbers at the bottom indicate the number of pixel composing the image. The scan size is 0.25 × 0.25 μm^2 ^for the left column and 0.125 × 0.125 μm^2 ^for the right column, while the intensity unit for both is 0.1 nm.

The denoising results of AFM imaging on biomembrane surfaces are shown in Figure 2. Two sets of images are arranged in two columns to indicate that the topographic measurements were independently performed with different experimental preparations and AFM setups. The representation of restored and noise images sharing the same intensity range with raw images is referred to additional file 1 Figure S2. We present here no details in AFM instrumentation or sample preparation, implying no need to know how this image was obtained for denoising. The top row presents the AFM raw images while the second and third rows illustrate the corresponding restored and noise images. The last row shows the Φ-image. Similar presentation for GTPase enzymes is presented in Figure 3.

We found that the Gaussian model was appropriate for fitting the intensity distribution of the central region, and for assigning an appropriate value to the center pixel, (*i_0_, j_0_*). A strong noise occurred at the center pixel usually leads to the appearance of heavy or notorious stripes in the image. The Gaussian model greatly reduced the number of pixels processed; one may notice much less pixels restored in the central region than in the off-center region. Naturally, one may speculate that there is more chance to recruit false noisy pixels in the off-center region. Recall that the FFT amplitude in the off-center region is much smaller and more homogeneous than that in the central region. The replacement of intensity values at false noisy pixels may not dramatically affect the image quality; that is the underlined rationale for dividing the candidate pixels into two regions.

In structural biology, the distribution of molecules is one feature of concern for AFM imaging. In general, observable individuality is a prerequisite prior to interpretation of molecular features for imaged objects. One may overlook important details simply because the entire image looks so dim due to the presence of stripe noises with very high intensity. The consequence of stripe removal can be evidenced by comparison of raw and processed images. First, the visibility of fine structures in the image was enhanced by the protocole. The left system in Figure 2 clearly demonstrates this effect. The restored image revealed that the dark areas in between bright segmental regions in the raw image were in fact distributed with granular particles. Second, the particle shape or cluster form became better observed. After noise reduction, stripes were eliminated or separated into short segments, and some brightly fused regions or islands were resolved into assemblies of individual granular particles; even the individual shape of background particles was noticeable.

Recently, enhancing image contrast by subtraction of a smoothed image from the raw image has been used for better visualizing the image objects [31]. The subtraction method is equivalent to extraction of edge features. In the present study, the purpose of denoising is to remove the noise prior to any other image processing and enhanced visibility on existent features in the image is a natural result.

### Noise vs. the image quality of AFM

For the same biological molecule but prepared in different conditions, the occurring pattern of noise is dissimilar as illustrated in Figures 2 and 3. Note that stripes can be due to horizontal or vertical noises [32]. In AFM surface measurements, vertical stripe is the major pattern of noise that intensely affects image quality. To a worse degree, their intensity distribution is not uniform, as seen in the raw image on the left of Figure 2. This complicates noise characterization and estimation. Likewise, these inhomogeneous stripes are also observable form the right noise image of Figure 3. In the right image of Figure 3 two observed dark bands are however due to scan lines misalignments along the height ordinate. *DeStripe *does not aim to remove such type of noise, yet it can be eliminated or reduced by flattening the image [31].

The noise images in Figures 2 and 3 present the noise components peeled off from the raw image. Comparing AFM images on different systems, the degree of noise removal by *DeStripe *was found to vary. This reflects in the distribution of potentially noisy pixels and the values in the Φ-image. From the intensity distribution of Φ-image, one may perceive the noisy pixels identified and the degree of intensity modification. The smaller intensity value in the Φ-image, the greater proportion of the spectrum amplitude is removed. The performance of *DeStripe *in denoising can be evaluated by visual inspection of the noise image. Our results show that the stripe noise pattern is very different from one system to the other. It implies that *DeStripe *is able to automatically tune the denoising performance. It is noteworthy that here all the images processed by *DeStripe *use the same set of parameter values; these values were chosen by trial and error such that the denoising can be effective for various images. Consequently, the only user-provided input is the raw image.

Taken together with restored images, we found that *DeStripe *under-denoises somewhere but over-denoises elsewhere in the raw image. On the one hand, it is trivial to diagnose a case of under-denoising if there is any stripe noise visible in the restored image. By lowering the heterogeneity threshold to include more candidate pixels in C_n_2, we found that the visible stripe noises can be further removed from the restored image (data not shown). We have also attempted to use lower values for restored intensities and some stripes diminished. On the other hand, if there is any non-stripe structure pattern in the noise image correlated with object features in the restored image, it may imply that *DeStripe *cut off too much the image intensity. In order to obtain the true surface measured by AFM, the structure pattern of noise image can be used as a guide for judging whether the denoising is appropriate. All the noise images presented here exhibit almost purely stripe noises, mainly in linear form. Indeed, a compromise between noise reduction and preservation of structural features remains challenging.

By comparing intensity range between restored and raw images, our results yield a smaller size for restored images than for the raw ones. Nevertheless, except for the left system in Figure 2 all other systems show comparable sizes for both images. From the results of the left system in Figure 2 the range of restored intensities is almost half that of the raw ones; it implies that near 50% of the intensity magnitude measured at some pixels by AFM is attributed to the noise. Certainly, those topographic measurements cannot represent a true value for the molecular surface; one can no longer consider these topographic measurements as true surface heights if heavy and bright stripe noises contaminate the image at such level. Consequently, the denoising is inevitable for better characterizing the surface feature of AFM images for a realistic biological system.

### Comparisons of denoising effects with Gwyddion

**Figure 4:**
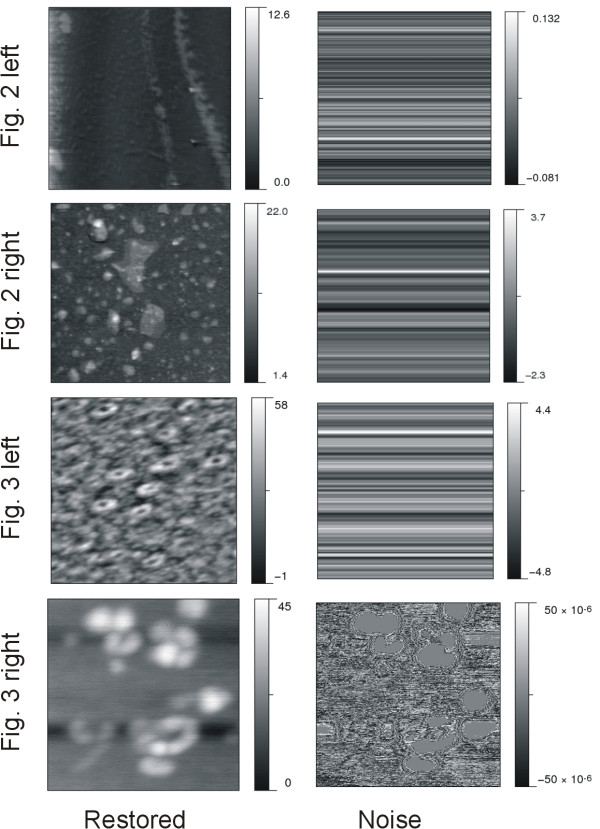
**Denoising effects on AFM images by the Gwyddion software**. The intensity value of the noise was obtained by subtracting the restored intensity from the raw measurement.

*Gwyddion *is open-source software for AFM image processing [33]. We applied the "correct lines" tool for comparing the denoising effects on high-density stripe removal. The results from *Gwyddion *are presented in Figure 4. By visual inspection, *Gwyddion *scarcely removed any noise from the image presented in row 4; this is observable either by comparing the restored and the raw images or from the intensity range of the noise image. As a matter of fact, the intensity values are essentially zero in the noise image on the right of Figure 3. The comparison reveals that *DeStripe *is more effective than *Gwyddion *on stripe reduction for AFM images. As a result, *DeStripe *manifests better the individuality of objects observed from the AFM image.

For topographic measurements, we assume that the noise is a positive quantity overlaid over the molecular surface; therefore the denoising effect should not yield restored images with greater intensity values than the raw measurements. In the present work, the processed images by *DeStripe *satisfy this important feature, revealing no other additive component imposed on the original image during the denoising procedure. This also reflects in the positive range of intensity in the noise images. In contrast, some noise intensities fall within the negative range in the denoising results from *Gwyddion*, see Figure 4. This indicates that *Gwyddion *may yield some restored intensities greater than the original data, thus creating new artifacts in the raw AFM image. Consequently, the two advantageous attributes, i.e., effective denoising and no extra artifact, make *DeStripe *superior to *Gwyddion *in improving the AFM image quality.

### Application to SEM image

**Figure 5:**
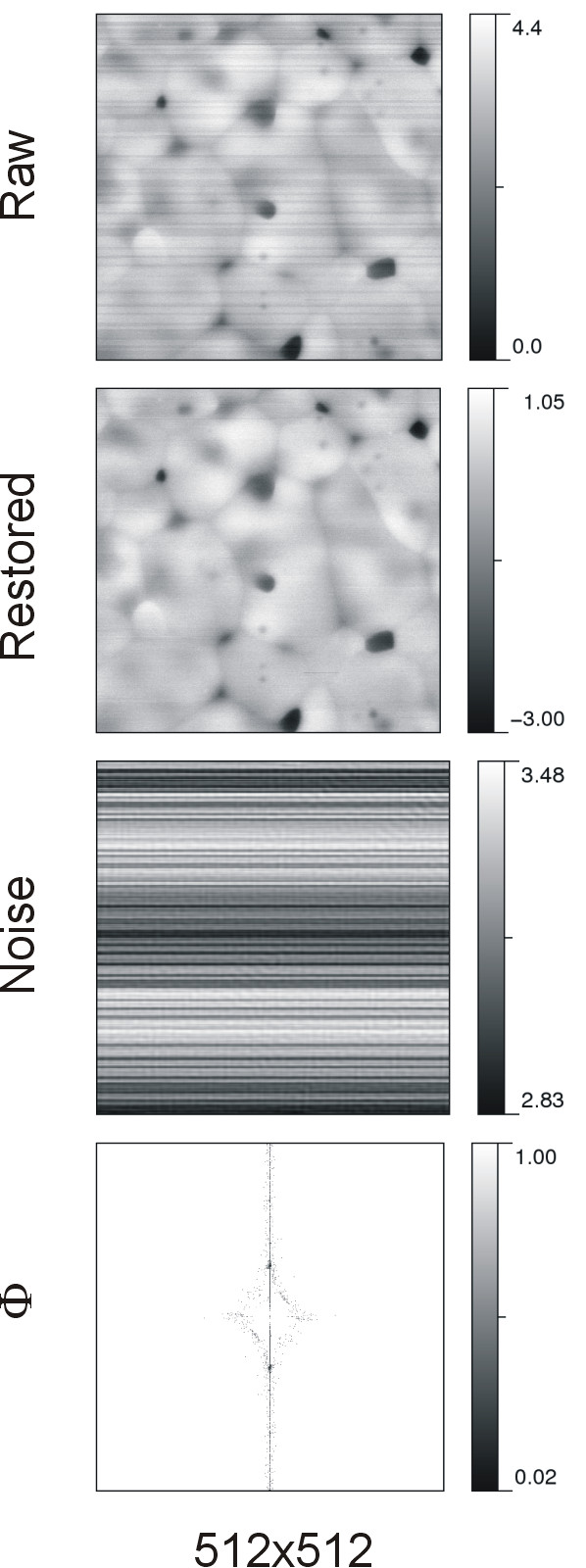
**SEM imaging on a sintered specimen of CeO_2 _(cerium oxide) at a sintering temperature of 1400°C for 2 hours and 50 minutes**. The image is composed of 512 × 512 pixels with each side measuring 70.55 μm; the intensity unit is 0.1 μm.

*DeStripe *performs denoising using an anisotropic Gaussian function to fit the spectral amplitude distribution close to the origin of the frequency domain. This modeling has been shown to provide an appropriate estimate of intensity for the origin of the frequency domain that is corrupted with noise. To further explore this aspect, we ran the program for a SEM micrograph. The results are presented in Figure 5. The originally measured image is seriously interfered with stripe noises, and we found that these severe stripes were mainly attributed to the contribution of the amplitude value at the origin. This ascertains that *DeStripe *strategy design is useful for images corrupted with heavy stripe noises. This image was processed using the same set of parameter values as for the AFM images.

## Conclusions

AFM emerges as a new nanotechnology for investigating the structure of biological systems with mesoscopic sizes, ranging from cellular morphology to single protein topography. Although this technique can probe bio-molecular surface down to a sub-nanometer scale, noise artifacts produced along with the measurements are also visible. We aimed to unveil the layer of noise from the image in order to witness the true topography beneath, and thus developed the automatic program *DeStripe*. By ripping off the noise contribution from the measured intensity, one obtains the surface heights closer to the true value and is able to inspect the surface features more easily; implicitly, the denoising processing enhances the molecular features of concern.*DeStripe *involves several methods for reducing the number of pixels for intensity restoration, including global sampling, non-local variance test, Gaussian fitting, and local CVAR test. Gaussian modeling revealed its superiority over conventional variance test in identifying the noisy pixels in the central region where intensity drastically changes. Moreover, the criterion of non-negative noise was insistently used for the reason that AFM imaging was directed for probing surfaces unexplored before; denoising simply for pleasing human eye is a false pursuit.

## Availability and requirements

• Project name: DeStripe

• Project server: http://biodev.cea.fr/destripe

• Operating system: Linux

• Programming language: Fortran 77 and C

• Any restriction to use by non-academics: license needed

## List of abbreviations

AFM: atomic force microscope; SEM: scanning electron microscope; SPM: scanning probe microscope; FFT: fast Fourier transform; CVAR: constrained variance; GTP: guanosine tri-phosphate.

## Authors' contributions

SWWC participated in the design of the study, carried out the software development, and drafted the manuscript. JLP participated in the design and coordinated the study. Both authors read and approved the final manuscript.

## Supplementary Material

Additional file 1 **DeStripe: supplementary figures**. The file contains two figures. Click here for file

## References

[B1] Binnig G Quate CF Gerber C Atomic force microscope Phys Rev Lett 1986 11 930 933 10.1103/PhysRevLett.56.930 10033323

[B2] Fechner P Boudier T Mangenot S Jaroslawski S Sturgis JN Scheuring S Structural information, resolution, and noise in high-resolution atomic force microscopy topographs Biophys J 2009 11 3822 3831 10.1016/j.bpj.2009.02.011 19413988 PMC2711429

[B3] Hörber JKH Miles MJ Scanning probe evolution in biology Science 2003 11 1002 1005 14605360 10.1126/science.1067410

[B4] Parot P Dufrene YF Hinterdorfer P Le Grimellec C Navajas D Pellequer JL Scheuring S Past, present and future of atomic force microscopy in life sciences and medicine J Mol Recogn 2007 11 418 431 10.1002/jmr.857 18080995

[B5] Ando T Uchihashi T Kodera N Yamamoto D Taniguchi M Miyagi A Yamashita H High-speed atomic force microscopy for observing dynamic biomolecular processes J Mol Recognit 2007 11 448 458 10.1002/jmr.843 17902097

[B6] Engel A Gaub HE Structure and mechanics of membrane proteins Annu Rev Biochem 2008 11 127 148 10.1146/annurev.biochem.77.062706.154450 18518819

[B7] Oesterhelt F Scheuring S High-resolution imaging and force measurement of individual membrane proteins by AFM Curr Nanosci 2006 11 329 335 10.2174/157341306778699347

[B8] Hoh JH Lal R John SA Revel JP Arnsdorf MF Atomic force microscopy and dissection of gap junctions Science 1991 11 1405 1408 10.1126/science.1910206 1910206

[B9] Muller DJ Schabert FA Buldt G Engel A Imaging purple membranes in aqueous solutions at sub-nanometer resolution by atomic force microscopy Biophys J 1995 11 1681 1686 10.1016/S0006-3495(95)80345-0 7612811 PMC1282071

[B10] Scheuring S Seguin J Marco S Levy D Robert B Rigaud JL Nanodissection and high-resolution imaging of the Rhodopseudomonas viridis photosynthetic core complex in native membranes by AFM. Atomic force microscopy Proc Natl Acad Sci USA 2003 11 1690 1693 10.1073/pnas.0437992100 12574504 PMC149894

[B11] Odorico M Teulon JM Bellanger L Vidaud C Bessou T Chen S-wW Quemeneur E Parot P Pellequer JL Energy landscape of chelated uranyl - antibody interactions by Dynamic Force Spectroscopy Biophys J 2007 11 645 654 10.1529/biophysj.106.098129 17449661 PMC1896262

[B12] Robert P Benoliel AM Pierres A Bongrand P What is the biological relevance of the specific bond properties revealed by single-molecule studies? J Mol Recognit 2007 11 432 447 10.1002/jmr.827 17724759

[B13] Neuman KC Nagy A Single-molecule force spectroscopy: optical tweezers, magnetic tweezers and atomic force microscopy Nat Meth 2008 11 491 505 10.1038/nmeth.1218 PMC3397402 18511917

[B14] Teulon JM Parot P Odorico M Pellequer JL Deciphering the energy landscape of the interaction uranyl-DCP with antibodies using dynamic force spectroscopy Biophys J 2008 11 L63 L65 10.1529/biophysj.108.141937 18790844 PMC2576404

[B15] Gaboriaud F Parcha BS Gee ML Holden JA Strugnell RA Spatially resolved force spectroscopy of bacterial surfaces using force-volume imaging Colloids Surf B Biointerfaces 2008 11 206 213 10.1016/j.colsurfb.2007.10.004 18023156

[B16] Walter NG Huang CY Manzo AJ Sobhy MA Do-it-yourself guide: how to use the modern single-molecule toolkit Nat Meth 2008 11 475 489 10.1038/nmeth.1215 PMC2574008 18511916

[B17] Müller DJ Helenius J Alsteens D Dufrêne YF Force probing surfaces of living cells to molecular resolution Nat Chem Biol 2009 11 383 390 19448607 10.1038/nchembio.181

[B18] Zhong Q Inniss D Kjoller K Elings V Fractured polymer/silica fiber surface studied by tapping mode atomic force microscopy Surf Sci Lett 1993 11 L688 L692 10.1016/0039-6028(93)90582-5

[B19] Gravel P Beaudoin G De Guise JA A method for modeling noise in medical images IEEE Trans Medical Imaging 2004 11 1221 1232 10.1109/TMI.2004.832656 15493690

[B20] Gonzalez RC Woods RE Digital image processing 2008 3 New Jersey: Pearson Education

[B21] Munch B Trtik P Marone F Stampanoni M Stripe and ring artifact removal with combined wavelet-Fourier filtering Opt Express 2009 11 8568 8591 10.1364/oe.17.008567 19434191

[B22] Aizenberg I Butakoff C Frequency domain median-like filter for periodic and quasi-periodic noise removal SPIE Proc Image Process 2002 11 181 191

[B23] Chan RH Ho C-W Nikolova M Salt-and-pepper noise removal by median-type noise detectors and detailpreserving regularization IEEE Trans Image Process 2005 11 10 1479 1485 10.1109/TIP.2005.852196 16238054

[B24] Koziol JA Intutive biostatistics 1995 New York: Oxford University Press

[B25] Pok G Liu J-C Nair AS Selective removal of impulse noise based on homogeneity level information IEEE Trans Image Process 2003 11 85 91 10.1109/TIP.2002.804278 18237881

[B26] Baierlein R Newtonian dynamics 1983 McGraw-Hill

[B27] Madsen K Nielsen HB Tingleff O Methods for non-linear least squares problems 2004 2 Technical University of Denmark

[B28] Lampert CH Wirjadi O An optimal nonorthogonal separation of the anisotropic Gaussian convolution filter IEEE Trans Image Process 2006 11 3501 3513 10.1109/TIP.2006.877501 17076408

[B29] Reimer L Hawkes PW Scanning electron microscopy: physics of image formation and microanalysis 1998 2 Springer

[B30] Trinh M-H Odorico M Bellanger L Jacquemond M Parot P Pellequer J-L Tobacco mosaic virus as an AFM tip calibrator J Mol Recognit 2011 11: 10.1002/jmr.1118 21504029

[B31] Kienberger F Pastushenko VP Kada G Puntheeranurak T Chtcheglova L Riethmueller C Rankl C Ebner A Hinterdorfer P Improving the contrast of topographical AFM images by a simple averaging filter Ultramicroscopy 2006 11 822 828 10.1016/j.ultramic.2005.11.013 16675120

[B32] Chi C-y Zhang J-x Liu Z-j Study on methods on noise reduction in a stripped image XXI ISPRS Congress, Youth Forum: 2008; Beijing 2008 The International Archives of the Photogrammetry, Remote Sensing and Spatial Information Sciences 213 216

[B33] Gwyddion - Free SPM (AFM, SNOM/NSOM, STM, MFM, ...) data analysis software http://gwyddion.net

