# Supplementary material for: DeStripe: frequency-based algorithm for removing stripe noises from AFM images
Source: BMC Struct Biol. 2011 Feb 1;11:7. doi: 10.1186/1472-6807-11-7 (PMC3749244; doi:10.1186/1472-6807-11-7)

# Additional file 1

# DeStripe: frequency-based algorithm for removing stripe noises from AFM images

# Shu-wen W. Chen, Jean-Luc Pellequer*

**Fig. S1:** Mapping of noisy pixel sets that were sequentially identified in the frequency domain, see Fig.1. In the order of increasing brightness: P_n_2, C_0_ (P_n_2+C_0_ = P_n_1) are presented in column 1, C_n_1, P_n_3, C_n_2 for column 2, and the final set of identified noisy pixels is shown in column 3.

**
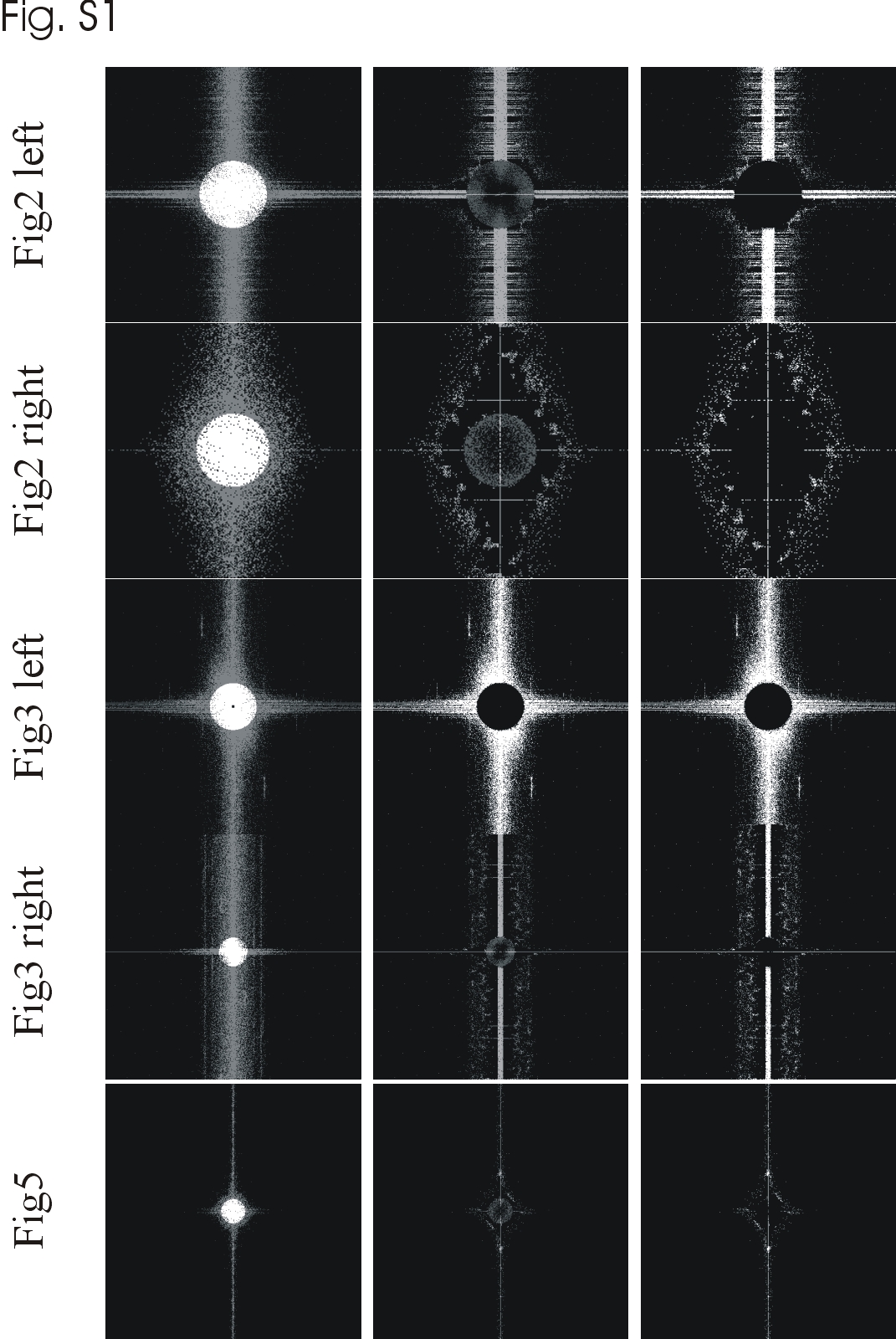
**

**Fig. S2:** Demonstrations of restored and noise images by *DeStripe* in accordance with the intensity range of raw images. The restored images have been offset to zero.


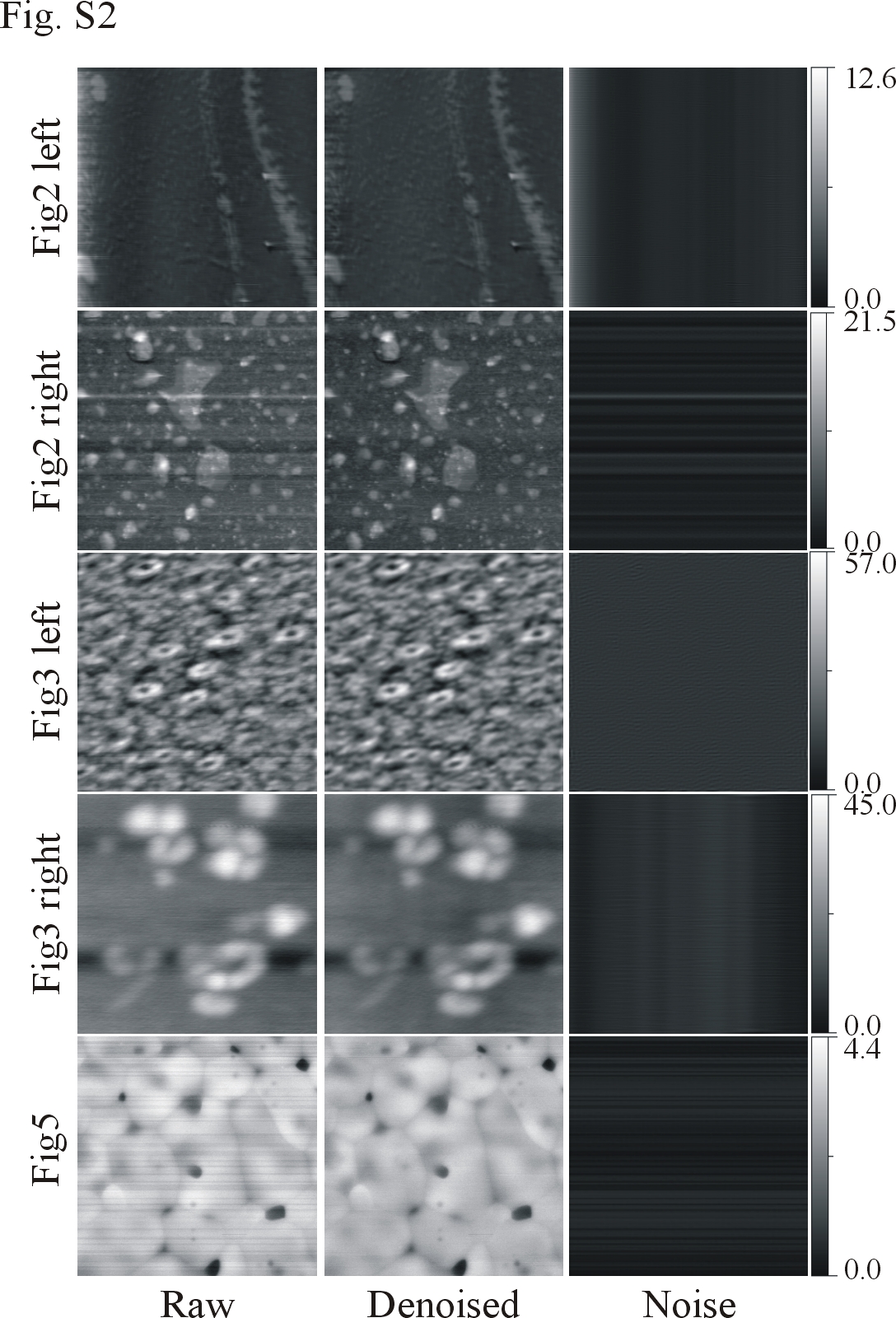

Supplement: Additional file 1 — DeStripe: supplementary figures. The file contains two figures. [file 1472-6807-11-7-S1.DOC]
